# Immunopathological characterization of ovarian teratomas associated with anti-N-methyl-D-aspartate receptor encephalitis

**DOI:** 10.1186/s40478-019-0693-7

**Published:** 2019-03-11

**Authors:** Aude Chefdeville, Isabelle Treilleux, Marie-Eve Mayeur, Coline Couillault, Géraldine Picard, Chloé Bost, Karima Mokhtari, Alexandre Vasiljevic, David Meyronet, Véronique Rogemond, Dimitri Psimaras, Bertrand Dubois, Jérôme Honnorat, Virginie Desestret

**Affiliations:** 1 Institut NeuroMyogène, Equipe Synaptopathies et Autoanticorps (SynatAc), INSERM U1217/UMR CRS 5310, Lyon, France; 2 University of Lyon, Université Claude Bernard Lyon 1, Lyon, France; 3 Department of Biopathology, Centre Leon Berard, Lyon, France; 4 INSERM 1052, CNRS 5286, Centre Leon Berard, Centre de Recherche en Cancérologie de Lyon, Lyon, France; 5 French Reference Center on Paraneoplastic Neurological Syndrome, Hospices Civils de Lyon, Lyon, France; 6 Raymond Escourolle Neuropathology Laboratory, Groupement Pitie-Salpetriere, AP-HP, Paris, France; 7 Department of Pathology, Groupement hospitalier Est, Hospices Civils de Lyon, Lyon, France; 8 Hôpital Neurologique Pierre Wertheimer, 59 Boulevard Pinel, 69677 Bron Cedex, France

**Keywords:** Ovarian teratoma, Anti-NMDAR encephalitis, Autoimmunity

## Abstract

Encephalitis with anti-NMDAR antibodies (NMDAR-E) is a severe autoimmune neurological disorder, defined by a clinical presentation of encephalitis and the presence of IgG targeting the GluN1 subunit of NMDA receptors in the CSF. An underlying ovarian teratoma is commonly associated with this autoimmune disease suggesting a role of the tumor in immunopathogenesis. In this study, we characterized the salient histopathological features of 27 ovarian teratomas associated with NMDAR-E (3 immature and 24 mature teratomas) and 40 controls without associated encephalitis. All but one NMDAR-E-associated teratomas contained a nervous tissue component, while less than 40% of control teratomas did (*p* < 0.001). GluN1 expression by teratomatous nervous tissue seemed to be more often glial in NMDAR-E teratomas than in control teratomas (73% vs. 29%, *p* < 0.05). Strikingly, 3 out of 24 NMDAR-E-associated mature teratomas contained neuroglial tissue exhibiting histopathological features of central nervous system neuroglial tumor, while such glioma-like features are exceptionally described in the literature on ovarian teratomas. Moreover, NMDAR-E associated teratomas differed from sporadic ovarian teratomas by consistent and prominent infiltration of the nervous tissue component by immune cells, comprised of T- and B-cells and mature dendritic cells organized in tertiary lymphoid structures, with IgG and IgA deposits and plasma cells in close contact to the neuroglial tissue.

These data demonstrate an association between massive infiltration of NMDAR-E-associated teratomas by immune cells and particular glial features of its neuroglial component, suggesting that this glial tissue might be involved in triggering or sustaining the anti-tumor response associated with the auto-immune neurological disease.

**Electronic supplementary material:**

The online version of this article (10.1186/s40478-019-0693-7) contains supplementary material, which is available to authorized users.

## Introduction

Encephalitis (E) with anti-NMDA receptor (NMDAR) antibodies (NMDAR-E) is a recently described severe autoimmune neurological disorder, defined by a clinical presentation of encephalitis and presence of IgG targeting the GluN1 subunit of the NMDAR in patients’ cerebrospinal fluid (CSF) [7]. An underlying neoplasm is found in 25 to 40% of patients, primarily in young females, and this associated tumor is an ovarian teratoma in 90% of the cases [2, 8, 29]. This strong association suggests a role of the tumor in the immunopathogenesis of the autoimmune disease. Histopathological studies reporting presence of neuroglial tissue expressing NMDAR in ovarian teratoma associated with NMDAR-E [7, 14, 27, 30] raised the hypothesis that immunization against the NMDAR might be triggered by NMDAR expression by teratoma neuroglial elements. However, later studies including sporadic teratomas without associated NMDAR-E showed that presence of an ovarian teratoma with neuroglial tissue expressing NMDAR is not sufficient to induce anti-NMDAR auto-immune response [14, 20, 27], suggesting tumor specificities in NMDAR-E patients. Moreover tumor-like features of the teratomatous neuroglial component have been reported in the recent literature [9, 14]. Furthermore, a marked intratumoral lymphoid infiltrate colocalizing with mature neuroglial elements is reported in a few patients with NMDAR-E but is yet to be fully characterized [6, 9, 14, 19, 27, 30]. In the present study, we compared ovarian teratomas associated with NMDAR-E to those in patients with sporadic teratomas in order to describe histological features characterizing these tumors.

## Methods

### Patient samples

To be included, patients had to meet the recognized criteria for NMDAR-E associated with the presence of IgG directed against NMDAR in the CSF [7, 29]. Cases of NMDAR-E referred to the French Reference Center for Autoimmune Encephalitis between September 2007 and November 2018 were identified, and among these those with associated ovarian teratoma and for whom formalin-fixed paraffin-embedded (FFPE) samples of the resected ovarian teratomas were available were included in the present study. Control cases (sporadic, without associated NMDAR-E) were all ovarian teratomas resected in 2013 at the gynecology department of the *Hôpital Femme-Mère-Enfant* (Lyon, France), which is the largest center in the region, and analyzed by the *Service d’Anatomo-pathologie* (Hospices Civils de Lyon, Groupement Hospitalier Est, Lyon). The control cases were not tested for the presence of anti-NMDAR antibodies in CSF or serum. However, these patients have not developed neurological symptoms evoking auto-immune encephalitis since 2013.

### Tumor pathology

Four μm-thick FFPE tissue sections were stained with hematoxylin-phloxine-saffron (HPS). A referent pathologist (IT) assessed the histological features of ovarian teratomas, identified their various mixed germ cell components, and analyzed the maturity of neuroglial elements when present. When an immature tissue with neural differentiation was present, grading of immature teratomas was performed according to the World Health Organization (WHO) classification [16]. All available slides of each tumor were examined at low magnification by IT and VD and the inflammatory infiltrates in contact or inside the nervous component were assessed semi-quantitatively as absent (0), low (+), moderate (++), or high (+++). Cases containing nervous tissue elements presenting the pathological features of neuroglial tumors were further examined by neuropathologists (KM, AV, DM) and were described using World Health Organization terminology for neuroglial tumors [18].

### Immunohistochemical investigation

Immunohistochemistry, developed with diaminobenzine, and counterstained with hematoxylin, was performed on serial sections using automated staining systems (Discovery XT and Benchmark XT; Roche, Meylan, France) detailed in the Additional file 1, except for the GluN1 subunit of NMDAR that was immunostained following a manual protocol (detailed in the Additional file 1). Inflammatory cells were characterized by performing immunostaining specific for B-cells (CD20+), T-cells (CD3+), and mature dendritic cells (DC-Lamp+). Neural elements within teratomas were analyzed using neuronal markers (neurofilament – NF, chromogranin A – ChromoA) and glial markers (GFAP, Olig2), completed by routine immunomarkers dedicated to glial tumor phenotyping (PS100, EMA, ATRX, IDH1, INA, and CD34). Manual multiparametric immunofluorescence staining was performed to detect IgG and IgA -producing cells in the GFAP+ glial component. Antibodies used and concentrations are detailed in the Additional file 1: Table S1 in Online Resource.

### DNA sequencing

Next-generation sequencing (NGS) was performed on the nervous component of one paraffin-embedded teratoma sample (case#5) as described in the Additional file 1. The following 10 genes were sequenced: *ATRX*, *BRAF*, *CDKN2A*, *HIST1H3B/C*, *H3F3A IDH1*, *IDH2*, *TERT*, and *TP53*. Chromosomic deletions on 7q, 7p, 9p 10p, and 10q, as well as 1p/19q co-deletion were also investigated.

### Statistical analysis

Statistical analysis were performed using the R software version R-3.4.1 (https://cran.r-project.org/). *P*-values for nervous tissue components, GluN1 expression, and presence of inflammatory comparisons were obtained using the Fisher’s exact test for contingency tables with Bonferroni correction for multiple testing. Inter-rater agreement was quantified by the Kappa (k) statistic.

## Results

### Histological characteristics of NMDAR-E associated teratomas

During the study period, a total of 286 cases of NMDAR-E were identified, among whom 57 (19.9%) had an associated ovarian teratoma; 27/57 (47.4%) formalin-fixed paraffin-embedded (FFPE) samples were available and included in the present study. A total of 40 control ovarian teratomas were also included, all were mature and multitissular. Among NMDAR-E ovarian teratomas, 24/27 were mature multitissular teratomas; the 3 others contained foci of immature neural tissues (neuroepithelial tubules and neural blastema) and were thus diagnosed as immature ovarian teratoma (two grade 2 and one grade 1; Table 1).

**Table 1:** Histological features of NMDAR-E associated ovarian teratomas

Case #	Teratoma Grading	CNT Cystic/Solid	PNT /ENT	Inflammatory infiltrates in contact with CNT	TLS in contact with CNT
1	Immature (Grade 1)	Yes/Yes	No/Yes	++	0
2	Immature (Grade 2)	Yes/Yes	Yes/No	+++	0
3	Immature (Grade 2)	No/Yes	Yes/Yes	+	0
4*	Mature	Yes/Yes	No/No	+	0
5*	Mature	No/Yes	Yes/No	+	1
6*	Mature	Yes/Yes	No/No	+	1
7	Mature	Yes/No	No/No	+	0
8	Mature	Yes/Yes	No/No	++	0
9	Mature	Yes/No	No/No	++	0
10	Mature	No/Yes	No/No	++	1
11	Mature	Yes/Yes	Yes/Yes	++	0
12	Mature	No/No	No/No	NA	NA
13	Mature	Yes/Yes	No/No	++	0
14	Mature	Yes/Yes	No/No	+++	1
15	Mature	Yes/No	Yes/No	++	0
16	Mature	Yes/No	Yes/Yes	++	0
17	Mature	No/Yes	No/No	+	0
18	Mature	Yes/Yes	No/Yes	++	1
19	Mature	Yes/Yes	Yes/No	+++	1
20	Mature	Yes/Yes	Yes/No	+	1
21	Mature	No/Yes	Yes/No	+++	1
22	Mature	No/Yes	No/No	++	1
23	Mature	No/Yes	No/No	++	1
24	Mature	Yes/No	No/No	++	1
25	Mature	Yes/No	No/No	+++	0
26	Mature	No/Yes	No/Yes	+	1
27	Mature	No/Yes	No/Yes	+++	1

NMDAR-E: Encephalitis with anti-NMDAR antibodies; CNT: central nervous system-like teratomatous tissue; PNT: peripheral nervous tissue; ENT: Enteric nervous tissue. * Cases presenting histological features resembling glioma; TLS: Tertiary Lymphoid Structures; NA: not applicable

#### Neural differentiation in NMDAR-E associated teratomas

**Fig. 1:**
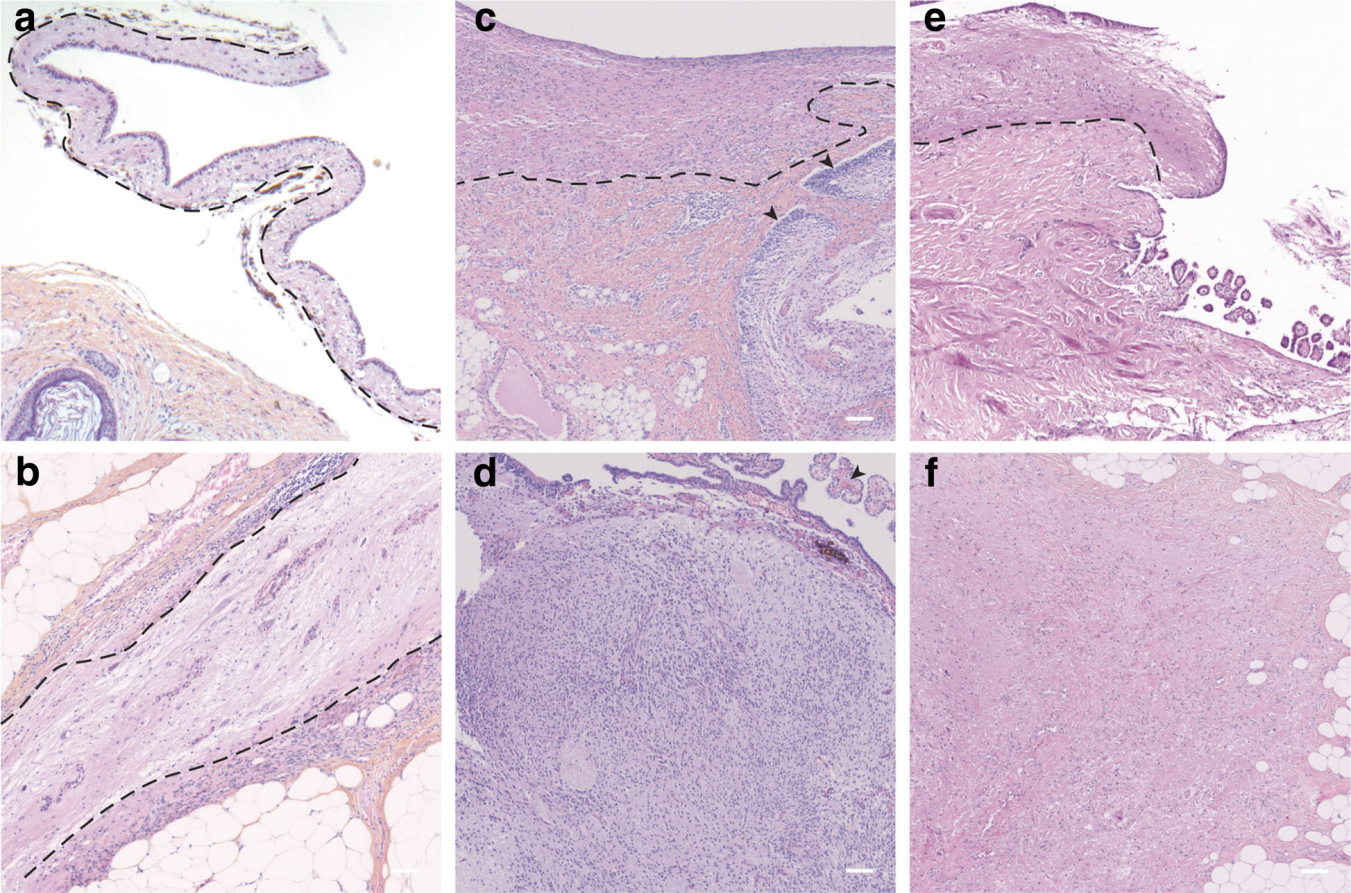
Central nervous tissue in NMDAR-E-associated and control ovarian teratomas. (a-b) Representative hematoxylin-phloxine-saffron (HPS) gross structure of NMDAR-E mature ovarian teratomas. In case (**a**), a strip of central nervous tissue (outlined, black-dotted lines) lined the inner wall of a cystic cavity. In case (**b**), the central nervous tissue (outlined, black-dotted lines) formed a solid mass between connective and adipose tissues (**c**-**d**) Representative HPS structure of immature ovarian teratomas associated with NMDAR-E. In (**c**), note the strip of central nervous tissue (outlined, black dotted lines) and the immature foci (arrowheads) with increased cellular density and neuroepithelial tubules. In case (**d**), the immature nervous contingent formed a solid mass lined with ependymal wall; note the choroid plexuses (arrowhead). **e**-**f** Representative HPS structure of control mature teratomas. The case (**e**) contained a strip of mature nervous tissue lining the wall of a cystic cavity (outlined, black dotted line). In the case (**f**), the solid mature nervous tissue was surrounded by connective and adipose tissue. Scale bar: 100 μm

Nervous tissue was present in all but one NMDAR-E associated teratomas (26/27, 96%) while only 15/40 (38%) of control teratomas included nervous tissue (*p* < 0.001). In the one NMDAR-E teratoma in which we did not observe a nervous tissue component, only half of the 2-cm tumor was embedded in paraffin. As expected, when present, the nervous component always contained a central nervous tissue-like differentiation (Fig. 1). Central nervous tissue appeared as a well demarcated solid mass (in 19/26 [73%] NMDAR-E and 9/15 [60%] control teratomas) or lining the inner wall of a cystic cavity (in 17/26 [65%] NMDAR-E and 12/15 [80%] control teratomas). Choroid plexuses were commonly observed in the central nervous tissue of NMDAR-E (12/26, 46%) and control teratomas (5/15, 33%). Peripheral and enteric nervous tissue could be also present (nerves and ganglia in 9/26 [34%] NMDAR-E-associated teratoma and 7/15 [46%] control teratomas; myenteric plexuses in 6/26 [23%] and 1/15 [6%]). When nervous tissue was present, there was no significant difference in the frequencies of central, peripheral, or enteric neural differentiation between NMDAR-E and sporadic ovarian teratomas (Table 2). These neuroglial elements were composed of glial cells that expressed GFAP or Olig2 (100% of cases), and neurons that expressed ChromoA (10/17 [59%] NMDAR-E and 6/12 [50%] control teratomas) or neurofilament F (7/17 [41%] NMDAR-E and 7/13 [54%] control teratomas; Table 3).

**Table 2:** Comparison between NMDAR-E-associated and control teratomas with nervous tissue

	NMDAR-E teratomas (*n* = 27)	Control teratomas (*n* = 40)	*p*-value
Nervous tissue, n (%)	26 (96)	15 (38)	5. 10^−7^***
Central	26/26 (100)	15/15 (100)	1
Solid	19/26 (73)	9/15 (60)	0.49
Cystic	17/26 (65)	12/15 (80)	0.47
Choroid plexus	12/26 (46)	5/15 (33)	0.51
Peripheral	9/26 (34)	7/15 (46)	0.51
Enteric	6/26 (23)	1/15 (6)	0.23
Teratomas displaying histological features of gliomas, n (%)	3/26 (12)	0/15 (0)	0.54
Inflammatory infiltrates in contact to central nervous tissue, n (%)	26/26 (100)	2/15 (13)	5. 10^−9^***
Tertiary lymphoid structures	13/26 (50)	1/15 (6)	0.006

***: significant after Bonferroni correction for multiple comparisons, p < 0.001

**Table 3:** Cell markers expressed by nervous tissue and inflammatory infiltrates in NMDAR-E-associated and control teratomas

	NMDAR-E teratomas (*n* = 27)	Control teratomas (*n* = 40)	*p*-value
Neuronal markers expression by neural elements when present, n (%)^a^	10/17 (58)	9/13 (69)	0.70
ChromoA+	10/17 (59)	6/12 (50)	0.71
NF+	7/17 (41)	7/13 (54)	0.71
GluN1 expression by neural element when present, n (%)^a^	18/22 (82)	11/14 (79)	1
Neuronal	12/22 (55)	9/14 (64)	0.73
Glial	16/22 (73)	4/14 (29)	0.015
Inflammatory infiltrates in contact to neural element when present, n (%)^a^	26/26 (100)	2/15 (13)	5. 10^− 9^***
B-cells (CD20+)	19/23 (83)	–	–
T-cells (CD3+)	22/23 (96)	–	–
Mature dendritic cells (DC-Lamp+)	11/24 (46)	–	–

^a^Percentages are calculated on the number of assessable cases. NF: neurofilament. ChromoA: chromogranin A

***: significant after Bonferroni correction for multiple comparisons, p<0.001

#### Neuroglial NMDAR expression in NMDAR-E associated teratomas

**Fig. 2:**
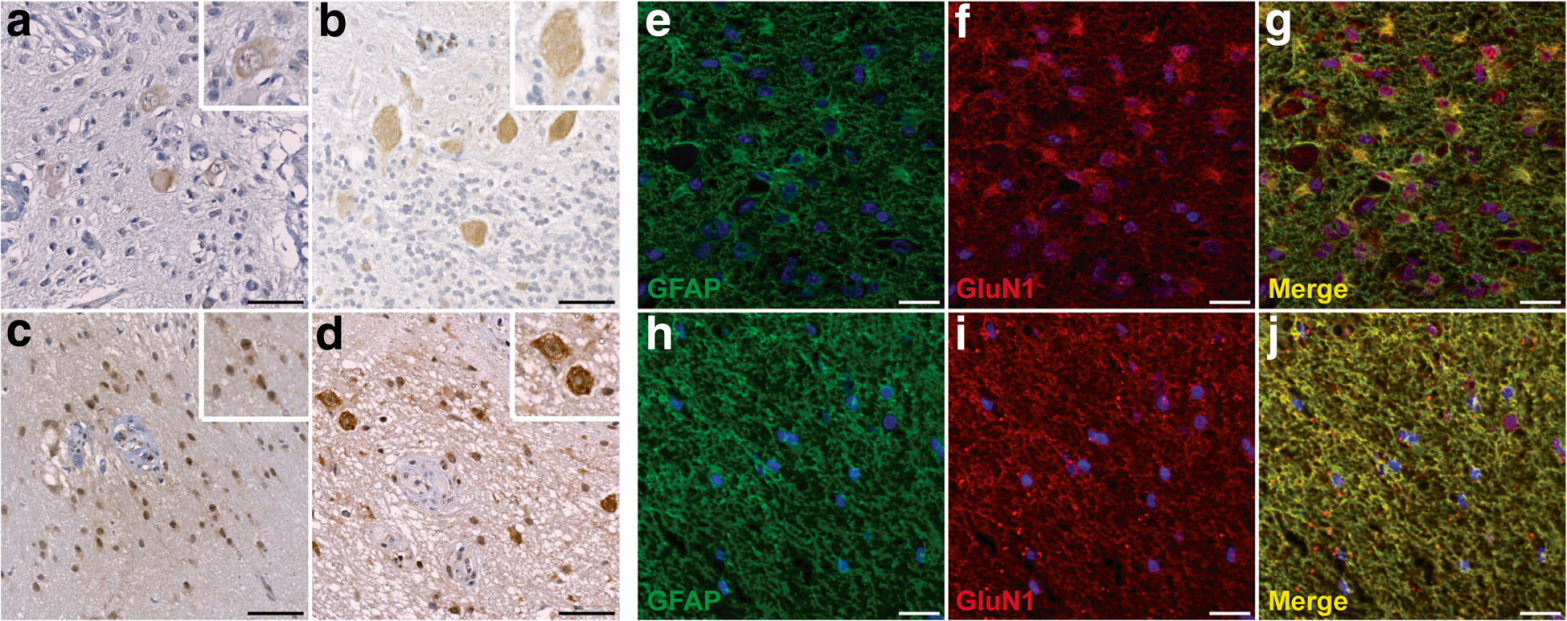
GluN1 expression by neuronal and glial cells in NMDAR-E associated and control ovarian teratomas. **a** Representative neuronal GluN1 immunostaining of ovarian teratomas associated with NMDAR-E. Neuronal expression of NMDAR subunit is detected in large ganglion cells (see cytoplasmic and weak nuclear staining on magnification). **b** Representative neuronal GluN1 immunostaining in the nervous tissue of a sporadic ovarian teratoma. Note the cerebellar-like organization with Purkinje-like cells expressing GluN1 (on magnification). **c** Representative glial GluN1 immunostaining of ovarian teratomas associated with NMDAR-E. GluN1 expression by astrocytic cells is cytoplasmic but also sometimes nuclear (on magnification). **d** Representative GluN1 immunostaining of sporadic mature ovarian teratomas with a nervous component. NMDAR subunit was expressed by neuropil, cell bodies of ganglion cells (arrows) and glial cells (on magnification). **e**-**g** Representative co-immunofluorescence stainings for GFAP (green) and GluN1 (red) in the central nervous tissue of NMDAR-E teratomas. **h**-**j** Representative co-immunofluorescence stainings for GFAP (green) and GluN1 (red) in the central nervous tissue of control teratomas. Scale bar: 50 μm

By immunostaining, NMDAR expression was detected in the nervous tissue of 18/22 (82%) teratomas associated with NMDAR-E and 11/14 (79%) control teratomas (Table 3). GluN1 was expressed by neurons (Fig. 2a-b) in respectively 12/22 (55%) NMDAR-E and 9/14 (64%) control tumors (Table 3), and by glial cells of astrocytic morphology both in NMDAR-E associated and control teratomas (Fig. 2c-d). Co-immunostaining with GluN1 and GFAP antibodies demonstrated that both proteins were co-expressed by astrocytes in NMDAR-E associated teratomas (Fig. 2e-g) and in control teratomas (Fig. 2h-j). This glial expression of GluN1 seemed to be more common in the nervous tissue of NMDAR-E associated ovarian teratomas (16/22 (72%) vs. 4/14 (28%); Table 3).

### Glioma-like features in NMDAR-E teratomas

**Fig. 3:**
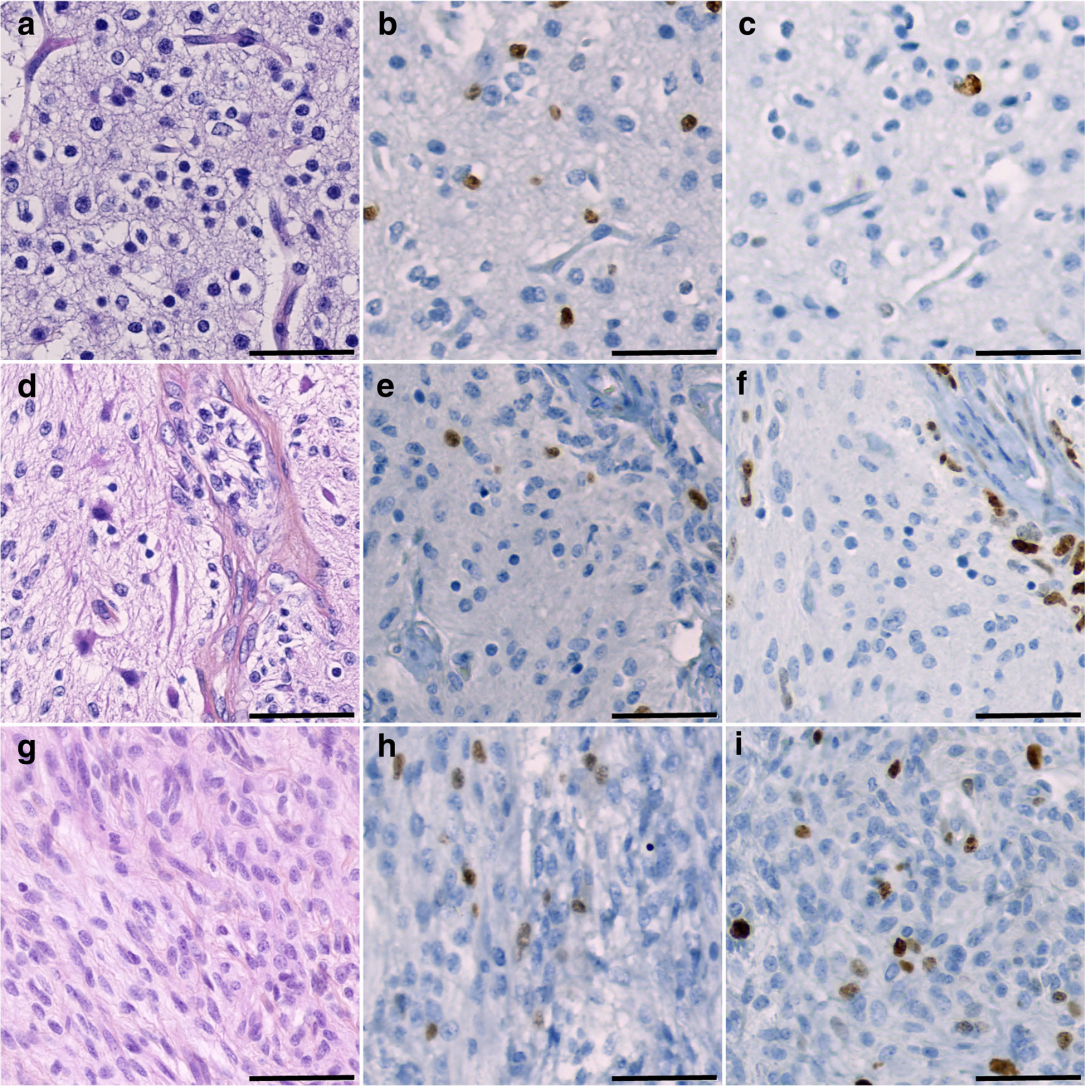
Histopathological phenotypes of NMDAR-E associated teratoma presenting histological features of gliomas. **a**-**c** Case #4. Hematoxylin-phloxine-saffron (HPS) staining (**a**) of the solid nervous tissue containing monotonous cells surrounded by perinuclear cytoplasmic halos (“fried-egg” oligodendrocytes-like cells) mixed with a vascular network consisting of short geometrically-arranged capillary segments, consistent with the histopathological observations of an oligodendroglioma. Some tumor cells showed positivity for Olig2 expression (**b**) but low Ki-67 proliferation index (**c**). **d**-**f** Case #5. HPS staining (**d**) of the neuroglial tissue showing increased cellular density, clusters of ganglion cells and aligned oligodendrocytes-like cells, consistent with the histological features of a ganglioglioma. Some tumor cells were found to be positive for Olig2 expression (**e**) and the Ki-67 proliferation index was increased in the perivascular area (**f**). **g**-**i** Case #6. HPS staining (**g**) of the nervous tissue with highly elevated cellular density and pleiomorphic or poorly differentiated cells, consistent with the histopathological aspect of a malignant glioma. Some tumor cells show positivity for Olig2 expression (**h**) and elevated Ki-67 proliferation index (**i**). Scale bar: 100 μm

Tumor-like areas with histopathological features of central nervous system (CNS) neuroglial tumor was observed in 3 cases of mature ovarian teratoma with NMDAR-E (patients #4, #5, and #6). The histopathological phenotypes of tumor-like nervous tissues of these 3 cases were respectively suggestive of an oligodendroglioma, a ganglioglioma, and a malignant glioma. Detailed examination of the nervous tissue from the teratoma resected from case #4 found a proliferation of monotonous cells with round and uniform nuclei surrounded by a perinuclear cytoplasmic halo mixed with a vascular network of short, geometrically-arranged, capillary segments, consistent with an oligodendroglioma not otherwise specified (NOS) (Fig. 3a). Tumor cells were positive for Olig2 (Fig. 3b), PS100 and ATRX, and negative for R132H-IDH1. Ki-67 proliferation index was < 5% (Fig. 3c). Histological investigation of the nervous tissue component from case #5 found elevated cellular density with presence of clusters of ChromoA-positive CD34-negative ganglion cells and alignments of oligo-like cells, evoking the histological features of a ganglioglioma (Fig. 3d). No bush-like astrocyte was detected with CD34 immunohistochemistry. In case #6, the nervous tissue component presented highly elevated cellular density and abundant pleomorphic cells (Fig. 3g), which were sometimes positive for Olig2 (Fig. 3h), PS100 or EMA. Ki-67 proliferation index was focally elevated at 8% (Fig. 3i). Necrosis, mitosis and microvascular proliferation were absent. In these 3 cases, no immature neural tissue was found. The genetic alterations commonly associated with gliomas were searched for using NGS analysis of the nervous component for case #5 (it was not possible for the 2 other cases because of technical failure); no mutation commonly associated with gliomagenesis was found. Medical NMDAR-E history and outcome of these 3 cases of NMDAR-E were classical and further described in the Additional file 1. There was no teratoma relapse, as expected in the case of mature teratoma.

### Characteristics of inflammatory infiltrates in NMDAR-E associated teratomas

**Fig. 4:**
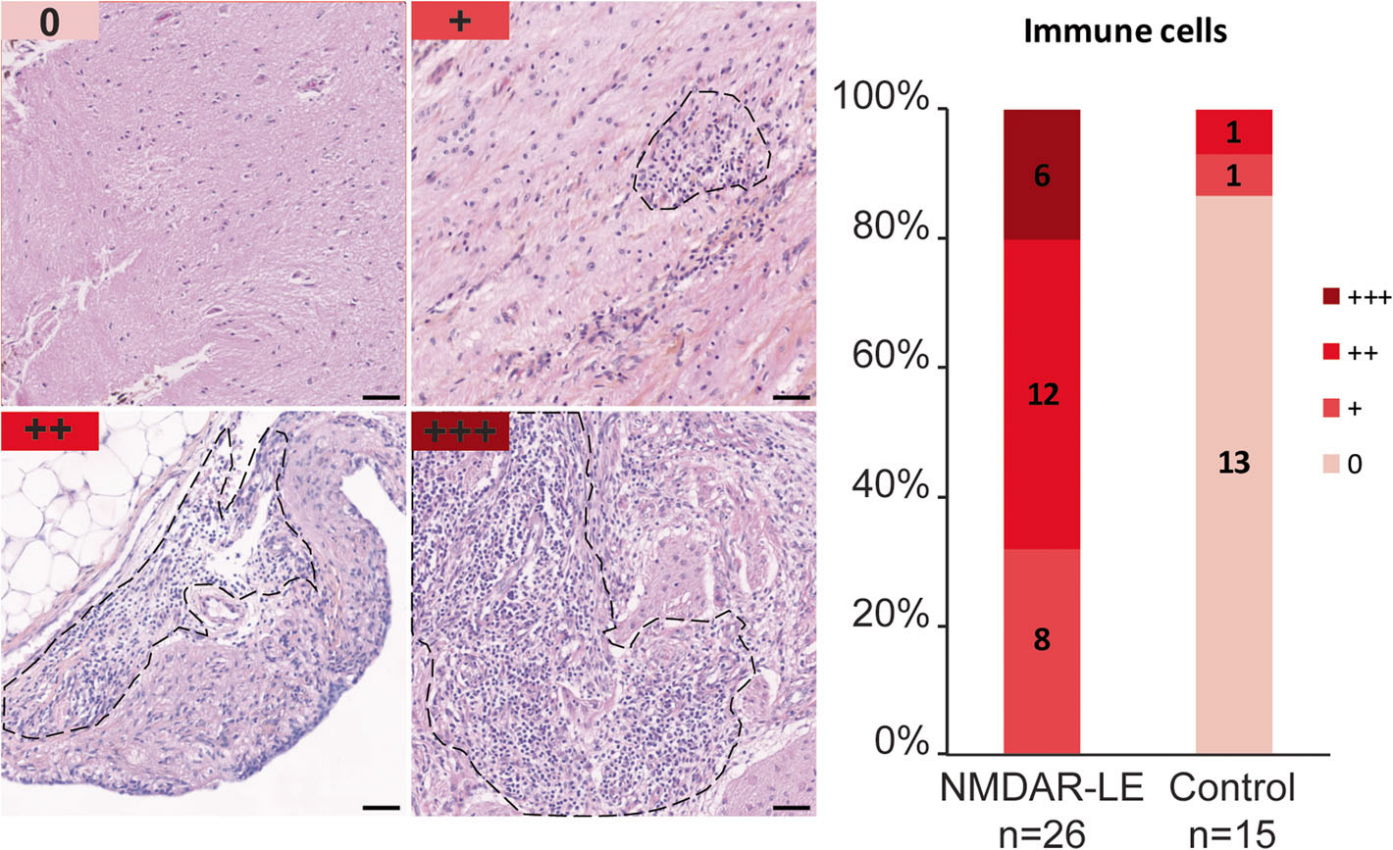
Inflammatory infiltrates in contact with nervous tissue in NMDAR-E-associated and control ovarian teratomas. Representative hematoxylin-phloxine-saffron (HPS) staining of teratoma nervous tissue with no (score 0), weak (score 1+), moderate (score 2+) or high (score 3+) immune cell infiltration (dotted). Scale bars: 50 μmn. Comparison of the semi-quantitative analysis of immune cell infiltrates between NMDAR**-**E associated (*n* = 26) and control (*n* = 15) ovarian teratomas with nervous tissue. Scale bar: 50 μm

**Fig. 5:**
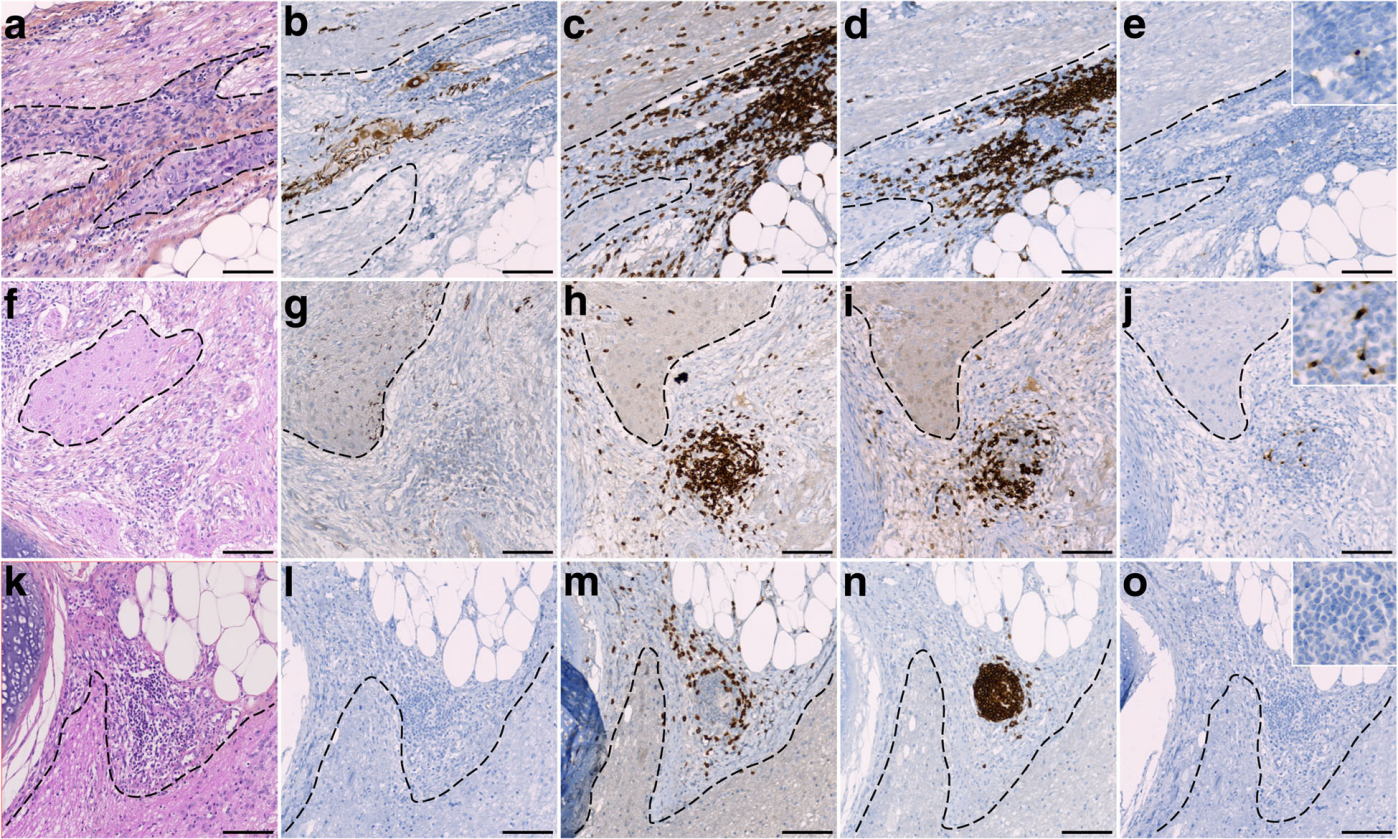
Characterization of the immune environment of the nervous tissue of NMDAR-E-associated ovarian teratomas. Representative nervous tissue of NMDAR-E teratoma infiltrated by immune cells and stained by hematoxylin-phloxine-saffron (**a**, **f**, **k**) or immunolabeled with the neuronal marker NF (**b**, **g**, **l**), the CD3 T-cell marker (**c**, **h**, **m**), the CD20 B-cell marker (**d**, **i**, **n**), or the mature dendritic cell marker DC-LAMP (**e**, **j**, **o**). **a**-**e** Diffuse inflammatory infiltrate closely adjacent to neural tissue (a, dotted area) containing NF-stained neurons (**b**) and composed of T-cells (**c**) and B-cells (**d**), with some mature dendritic cells (**e**, see higher magnification in the upper right corner). (**f**-**j**) Tertiary Lymphoid Structure (TLS) next to a focus of neuroglial tissue (**f**, dotted area) containing some NF positive neurites (**g**), with segregated B- and T-cells (**h**, **i**) and mature dendritic cells (j, see higher magnification in the upper right corner). **k**-**o** Lymphoid infiltrates close to a foci of neuroglial tissue (**k**, dotted area) without NF expression (**l**) and composed of segregated T-cells (**m**) and B-cells (**n**). Note the absence of mature dendritic cells (**o**, see higher magnification in the upper right corner). Scale bars: 50 μm

**Fig. 6:**
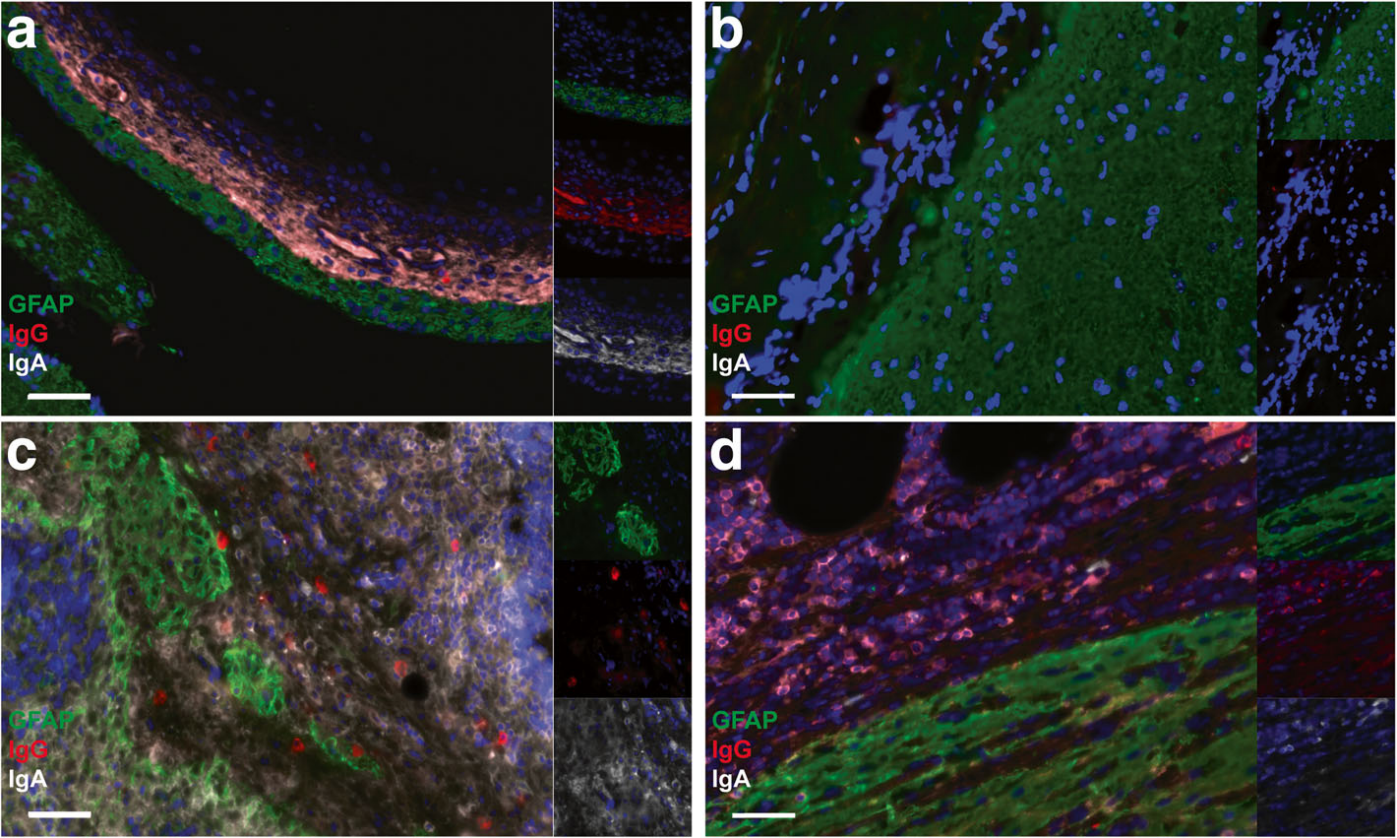
IgG and IgA deposits and producing cells in NMDAR-E associated teratomas. **a** Representative GFAP/IgG/IgA immunofluorescence (IF) staining of a NMDAR-E associated teratoma showing IgG (in red) and IgA (in white) deposits along a GFAP+ strip of neuroglial tissue (in green). Hoechst was used to visualize nuclei (blue) and pictures on the right correspond to individual channels. **b** Representative GFAP/IgG/IgA IF staining of a control teratoma without IgG nor IgA deposits in contact with GFAP+ neuroglial tissue. **c** GFAP/IgG/IgA IF staining of a NMDAR-E associated teratoma focused on GFAP+ neuroglial islands surrounded by immune cells and diffuse IgA deposits (white) and some isolated IgG+ cells (red). Hoechst was used to visualize nuclei (blue) and pictures on the right correspond to individual channels. **d** GFAP/IgG/IgA IF staining of a NMDAR-E teratoma showing individualized IgA+ cells (white) and IgG+ (red) in contact with GFAP+ neural elements (green) in a NMDAR-E associated teratoma. Scale bars: 50 μm

An immune cell infiltration closely adjacent to the neuroglial tissue was found in all 26 cases of NMDAR-E while only in 2/15 control teratomas (*p* < 0.001). Among NMDAR-E teratomas, 65% (18/26) displayed moderate to high inflammatory infiltrates (score of 2 or 3; Fig. 4; inter-rater agreement *k* = 1). There was no relationship between these immune cell infiltrates and the phenotype of the nervous tissue component (central, peripheral or enteric). These inflammatory infiltrates were composed of CD3+ T-cells and CD20+ B-cells (Fig. 5). In 50% of NMDAR-E associated cases, immune cell infiltrates in the nervous tissue component were organized in Tertiary Lymphoid Structures (TLS) with segregated T and B cell zones (13/26 (50%) vs. 1/15 (6%) control teratomas, *p* = 0.006; Fig. 5). Mature dendritic cells (DC-LAMP+), an essential component of TLS [13], were detected in T-cell-rich areas in 46% of cases with NMDAR-E associated ovarian teratoma (12/26; Fig. 5j). Multiplex immunofluorescence staining revealed the presence of diffuse IgG and IgA deposits and individualized IgG+ or IgA+ plasma cells in contact with the neuroglial tissue of 17/19 (89%) of NMDAR-E associate teratomas while only 3/15 (20%) control teratomas exhibited some weak IgG or IgA deposits without individualized stained cells (Fig. 6).

## Discussion

This histopathological study emphasizes the specificities characterizing ovarian teratomas associated with NMDAR-E. Notably, all but one NMDAR-E associated ovarian teratomas contained a nervous tissue component, while only just over a third of control ovarian teratomas did so. Furthermore, expression of the NMDAR GluN1 subunit by the teratomatous nervous tissue was significantly more often glial in NMDAR-E teratomas than in control teratomas. Another striking particularity was that among these 27 mature teratomas, 3 contained neuroglial tissue exhibiting histopathological features of CNS neuroglial tumor, while it was exceptionally reported in the literature on sporadic ovarian teratomas ([1, 3, 10, 15, 24, 25, 31–34] the histological classification of these 20 cases is summarized in Additional file 1: Table S2 in Online Resource). We also confirmed the particular immune environment of the nervous tissue of these teratomas that exhibited massive inflammatory infiltrates.

Although the frequency of immature teratomas among NMDAR-E associated cases herein is lower than that reported in the previously published case series [6, 9, 30], 14% of all reported cases (including those herein) are immature which is much greater than sporadic ovarian teratomas; only 3% of the latter are diagnosed as immature, mostly on the presence of immature neural tissue. [4, 22]. This difference could be related to ovarian teratomas of NMDAR-E patients more frequently containing neural tissues that have de facto the potential to be immature; neural tissue was found in all but one ovarian teratoma from patients with NMDAR-E but only just over a third of controls herein, and 30% of mature ovarian teratomas described in the literature [4, 21]. This suggests that the presence of nervous tissue may be essential to trigger the breaking of immune tolerance against NMDAR associated with encephalitis. Interestingly, the expression of this receptor in the nervous component of NMDAR-E teratoma has been detected in some cases by immunofluorescence using patient serum IgG [30] or immunohistochemistry against the GluN1 subunit [14, 27] and the GluN2B subunit of NMDAR [27]. Furthermore, using a commercial antibody we confirmed herein the positivity of teratoma nervous tissues for the GluN1 subunit of NMDAR in more than 80% of NMDAR-E teratomas of this large cohort. We also, however, report GluN1 expression in the same proportion of control teratomas with a neural differentiation, confirming that the mere ectopic expression of GluN1 is not per se sufficient to trigger the cross-immune reaction leading to neurological symptoms. This is supported by the report of GluN1 and GluN2B expression in epithelial ovarian carcinoma without occurrence of paraneoplastic NMDAR-E [23]. A possible explanation could be related to the NMDAR subunit composition profile or the cellular phenotype of cells expressing NMDAR. Herein, GluN1 was expressed both by neuronal and glial cells composing the nervous component of ovarian teratomas associated or not with NMDAR-E; neuronal expression is expected but glial expression is noteworthy, even if NMDAR expression by human astrocytes has been described [5, 17]. This glial GluN1 expression was more frequent in NMDAR-E associated teratoma than in sporadic cases, which might suggest that type of neural cells expressing the antigen NMDAR could be involved in the outbreak of NMDAR-E.

The present study establishes that other neuroglial features also distinguish NMDAR-E teratomas from sporadic ovarian teratomas. For instance, in three patients the mature nervous component presented tumor-like areas characterized by histological features of CNS tumors [18]. Such foci of nervous tissue forming a histological pattern reminiscent of glioblastoma can be observed in ovarian teratoma [28]. However, less than 25 cases of mature ovarian teratoma with nervous tissue exhibiting histological features of glioma (mostly glioblastoma and oligodendroglioma) have been described since 1960 [1, 3, 10, 15, 24, 25, 31–34], making these extremely rare events. The presence of a singular neuronal component in NMDAR-E teratomas has been previously suggested by the report of “abnormal neuronal elements” in 3 cases of mature ovarian teratomas [9]. Consistently, Iemura et al. have recently reported an “abnormal monotonous appearance” of the mature neuroglial tissue of 4 NMDAR-E teratomas with densely aggregated small neurons [14]. Herein, we precisely characterized these previously reported histological phenotypes that were consistent with neuroglial tumors; we observed obvious neuroglial changes that were not limited to the presence of “dysplastic or monotonous neurons”, but which also included gliomatous features, characterized by increased glial cell cellularity and glial marker expression, and clearly distinct from reactive abnormalities often observed in teratomas [9]. In one case, we further investigated the molecular phenotype of this glioma-like component. No gliomatous mutations were detected in this ganglioglioma-like proliferation, suggesting that adult CNS gliomagenesis pathways might not be involved in the glioma-like phenotype of the teratomatous tissue.

Besides these tumoral specificities, NMDAR-E-associated ovarian teratoma were also characterized by a massive and systematic infiltration of immune cells in close contact with nervous tissue component. Dense inflammatory infiltrates around neural tissue have been previously shown in NMDAR-E teratomas [6, 9, 14, 19, 27]. These lymphoid aggregates are composed of segregated B- and T-cells and sometimes organized in reactive tertiary lymphoid structures [6, 19]. We further revealed that immune infiltrates in NMDAR-E associated teratoma frequently contained mature dendritic cells in the vicinity of nervous tissue. These features are characteristics of tertiary lymphoid organs, that have already been reported in other tumors [13] and are believed to be generated as a result of local antigen presentation in a context of chronic inflammation and to perpetuate adaptive immune responses providing local source for antibody production [12]. Herein, we observed immunoglobulin deposits and immunoglobulin secreting cells in direct contact with the nervous tissue of NMDAR-teratomas, while previous studies had already detected plasma cells in NMDAR-teratomas [19, 32]. The presence of the B-cell response effectors in the tumor associated with B-mediated immunity supported the idea that in young females with NMDAR-E and ovarian teratoma, the tumor triggers the anti-NMDAR immune reaction. In particular, the detection of intra-tumoral IgA is consistent with the existing correlation between the presence of an associated teratoma and anti-NMDAR IgA in the CSF of patients with NMDAR-E [11]. All these data strongly suggest a link between the tumor immune environment and the CNS humoral auto-immunity directed against the onconeural antigen NMDAR. Recently, Makuch et al. demonstrated this link by showing that NMDAR ovarian teratoma tissue contained B cells which can produced IgG directed against GluN1 in culture [19]. We previously reported that genetic alteration of onconeural antigens, including gains and mutations, can play a major role in the induction of the immune response associated with paraneoplastic neurological syndromes [26]. Neoantigen production due to such genetic alterations in NMDAR coding genes might be also a potential mechanism leading to this singular anti-tumor immune response characterized by the intratumoral presence of B- and T-cell response effectors of the auto-immune disease.

Further molecular characterization of the expressed NMDAR antigens and gene expression profiling of tumor infiltrating immune cells in NMDAR-E teratoma are needed. This will require microdissection to improve the sampling of nervous tissue and infiltrates, and the prospective collection of fresh resected tumors to obtain a better quality of extracted DNA/RNA and to allow flow cytometry analysis of infiltrating immune cell population, as previously done in the cystic aspirate from one NMDAR-E teratoma by Makuch et al. [19].

In conclusion, the present study finds that the particular immune environment of the neuroglial tissue in NMDAR-E teratomas is associated with teratoma specificities. These histological features are unlikely to be sufficient per se to increase the immunogenicity of this ectopic nervous tissue; further investigation of teratoma genetic alterations is required to identify the molecular triggers of the immune tolerance breakdown leading to the auto-immune disease.

## Additional file

Additional file 1: Supplemental Methods: Immunohistochemistry (IHC) study. DNA sequencing. Supplemental Results: Clinical description of NMDAR-E cases presenting glioma-like feature teratomas. Supplemental Tables: **Table S1.** Antibodies used for immunohistochemistry and immunofluorescence stainings. **Table S2.** Reported cases of mature ovarian teratoma containing nervous tissue presenting histological features of glioma. (DOCX 34 kb)
