# Supplementary material for: Immunopathological characterization of ovarian teratomas associated with anti-N-methyl-D-aspartate receptor encephalitis
Source: Acta Neuropathol Commun. 2019 Mar 11;7:38. doi: 10.1186/s40478-019-0693-7 (PMC6410529; doi:10.1186/s40478-019-0693-7)
Supplement: Supplementary file 1 — Supplemental Methods: Immunohistochemistry (IHC) study. DNA sequencing. Supplemental Results: Clinical description of NMDAR-E cases presenting glioma-like feature teratomas. Supplemental Tables: Table S1. Antibodies used for immunohistochemistry and immunofluorescence stainings. Table S2. Reported cases of mature ovarian teratoma containing nervous tissue presenting histological features of glioma. (DOCX 34 kb) [file 40478_2019_693_MOESM1_ESM.docx]

**Electronic Supplementary Material**

**Online Resource**

**Table of contents**

[**Supplemental Methods** 2](#_Toc530933685)

[**Immunohistochemistry (IHC) study** 2](#_Toc530933686)

[**DNA sequencing** 2](#_Toc530933687)

[**Supplemental results** 4](#_Toc530933688)

[**Clinical description of NMDAR-E cases presenting glioma-like feature teratomas** 4](#_Toc530933689)

[**Supplemental Tables** 6](#_Toc530933690)

[**Table S1:** Antibodies used for immunohistochemistry and immunofluorescence stainings 6](#_Toc530933691)

[**Table S2:** Reported cases of mature ovarian teratoma containing nervous tissue presenting histological features of glioma 7](#_Toc530933692)

# **Supplemental Methods**

## **Immunohistochemistry (IHC)**

The antibodies (Abs) used and dilutions are summarized in Table S1.

*DAB chromogen immunohistochemistry for classical diagnostic makers*

IHC staining for immune cell markers (CD3, CD20, DC-LAMP) was performed using a routine protocol on a Discovery XT (Roche, Meylan, France) in the Biopathology department of the Centre Léon Bérard (Lyon, France). IHC staining for tumor cells markers (ChromoA, neurofilament, Ki-67, GFAP, Olig2, PS100, EMA,CD34, IDH1) was performed using a routine protocol on a BenchMark XT; Ventana (Roche, Meylan, France) in the Pathology Department of Groupement Hospitalier Est, Hospices civils de Lyon, Lyon, France.

*DAB chromogen immunohistochemistry against the GluN1 subunit of the NMDA receptor*

Four µm-thick FFPE tumor sections were deparaffinized in xylene and rehydrated in decreasing concentrations of ethanol, followed by antigen retrieval in 0.01M citrate buffer (pH=6). After blocking in 1% NGS, 1% BSA, 0.3% Triton X-100 in PBS, sections were incubated with the primary antibody (anti-GluN1, clone R1JHL, Merck Millipore, Molsheim, France) overnight at +4°C, thoroughly washed, then incubated with a biotinylated secondary antibody. Staining was developed using DAB chromogen (Vectastain ABC Kits, Vector Laboratories, Burlingame, CA USA). Slides were mildly counterstained in hematoxylin, dehydrated and mounted in DePeX (Thermo Fischer Scientific, Waltham, MA USA). A section of human cerebellum was used as a positive control, and primary antibodies were omitted in negative control.

*Immunofluorescence (IF) immunostaining*

Teratoma sections were deparaffinized and rehydrated, followed by antigen retrieval in 0.01M citrate buffer (pH=6). After blocking in 1% NGS, 1% BSA, 0.3% Triton X-100 in PBS, sections were incubated with primary antibodies (Table S1) overnight at +4°C, thoroughly washed and then incubated with a secondary antibody (Thermo Fischer Scientific, Waltham, MA USA) for 1h at room temperature. Fluorescence was developed using streptavidin coupled to a fluorochrome. Autofluorescence was eliminated by incubation with 0.1% Sudan Black B (Merck Millipore, Molsheim, France) in 70% ethanol. Nuclei were stained with DAPI and slides were mounted with FluorSave (Merck Millipore Molsheim, France. A section of human cerebellum was used as a positive control, and primary antibodies were omitted in negative control.

## **DNA sequencing**

Genomic DNA was extracted from the nervous tissue component of a paraffin-embedded teratoma sample. DNA library was prepared using the Kapa DNA Library Preparation Kit (Roche, Bale, Switzerland). Hybridization was performed at 47°C over 72 hours with primers designed using the SeqCapEZ (Roche, Bale, Switzerland) and hybridized sequences were isolated following the manufacturer’s protocol. After amplification, next generation sequencing was performed using the NextSeq500 from Illumina according to manufacturer’s instructions (Illumina, San Diego, CA USA).

# **Supplemental results**

## **Clinical description of NMDAR-E cases presenting glioma-like feature teratomas**

***Case #4***

A 38-year-old women presented with gait instability and diplopia. A prodromal episode of flu-like symptoms and headaches a month before initial symptoms was reported, otherwise medical history was unremarkable. Brain MRI was normal. The patient was confused, agitated, and presented signs of cognitive impairment. Fluctuating levels of consciousness and dysautonomia motivated her transfer to the intensive care unit (ICU), and later in the course of the disease she also developed seizures and dyskinesia. The patient improved after resection of a teratoma on the left ovary and treatment with intravenous immunoglobulin (IVIg) and corticosteroids. Neurological recovery was complete after 4 months and the patient remained symptom-free at the 2-years follow-up. Gross examination of the resected mass found a multi-tissular mature ovarian teratoma containing bones, fat, neuroglial tissue, and choroid plexuses. Histological investigation identified a proliferation of monotonous glial cells with round and uniform nuclei surrounded by a perinuclear cytoplasmic halo mixed with a delicate branching network of capillaries consistent with an oligodendroglioma.

***Case #5***

A 22-year-old women was admitted to hospital with abnormal movements, generalized seizures, dysautonomia, and central hypoventilation necessitating transfer to the ICU and ventilatory support. She had antecedents of behavioral and psychiatric disorder treated with neuroleptics. Initial MRI was normal but electroencephalography showed abnormalities suggestive of encephalopathy. Pelvic echography revealed the presence of a mass on the left ovary and a dermoid cyst was resected. The patient was treated with 6 courses of IVIg and corticosteroids. Neurological examination was normal after 6 months. Histological examination of the cyst found that it contained bone, fat, skin, and neural tissue. In the neural component, elevation of the cellular density, with clusters of ganglion cells and proliferation of aligned oligodendrocytes consistent with the histologic features of a ganglioglioma was observed.

***Case #6***

A 16-year-old girl initially presented with behavioral disorder, agitation, and disinhibition. Her condition worsened and she developed movement disorder, confusion, catatonia, mutism, fluctuating levels of consciousness, and dysautonomia. The patient was initially treated by corticosteroids followed by IVIg and plasma exchange, and underwent surgical resection of a mass on the right ovary. Upon diagnosis of NMDAR-E, treatment with second-line immunotherapy (rituximab and cyclophosphamide) was started. The patient responded to treatment and was completely free from symptoms at the 12 months follow-up. Macroscopically, the excised mass measured 4 cm in diameter. Histopathological investigation found a multi-tissular mature teratoma, containing skin, bone, fat, a tooth, choroid plexuses and neural tissue with strong transformations, with highly elevated cellular density and abundant pleomorphic cells and focally elevated proliferation index consistent with histological findings expected in a malignant glioma.

# **Supplemental Tables**

## **Table S1:** Antibodies used for immunohistochemistry and immunofluorescence stainings

| **Antibody** | **Provider** | **Catalog #** | **Dilution** | **Cell type stained** |
| --- | --- | --- | --- | --- |
| CD3 | Roche | 2GV6 | Pre-diluted | T lymphocytes |
| CD20 | Dako | L26 | 1:200 | B lymphocytes |
| Chromogranin A | Dako | DAK-A3 | 1:100 | Neuron (perikaryon), neuroendocrine cells |
| DC-Lamp | Dendritics | 1010E1.01 | 1:100 | Mature dendritic cell |
| GFAP | Dako for IHC  BD Biosciences for IF | Z0334  556330 | 1:500  1:500 | Astrocytes |
| GluN1 | Millipore | R1JHL | 1:250 for IHC | NMDAR- GluN1 subunit |
|  |  |  | 1:100 for IF |  |
| IgA | Dako |  | 1:200 | IgA deposits and IgA-producing cells |
|  | Southern Biotech |  | 1:500 | IgG deposits and IgG-producing cells |
| Ki-67 | Dako | MIB1 | 1:100 | Proliferating cells |
| Neurofilament | Dako | 2F11 | 1:100 | Neuron (axon) |
| Olig2 | Epitomics | EP112 | 1:400 | Oligodendrocytic lineage |
| PS100 | Dako | polyclonal | 1:100 | Cells derived from the neural crests |
| CD34 | Dako | QBEnd-10, | 1:50 | Progenitor cells |
| EMA | Dako | E29 | Pre-diluted | Normal and neoplastic epithelial cells |
| IDH1 | Dianova | DIA-H09 | 1:100 | Low grade and secondary high grade gliomas |

## **Table S2:** Reported cases of mature ovarian teratoma containing nervous tissue presenting histological features of glioma

| **Author, Year** | **Number of cases** | **Classification** |
| --- | --- | --- |
| Berger and Pochaczevsky, 1969 [1] | 2 | Astrocytoma |
| Kleinman et al., 1993 [15] | 7 | Glioblastoma |
| Den Boon et al., 1999 [2] | 1 | Glioblastoma |
| Yadav et al., 1999 [33] | 1 | Glioblastoma |
| Zannoni et al., 2002 [34] | 1 | Oligodendroglioma |
| Opris et al., 2009 [24] | 1 | Oligodendroglioma (low grade) |
| Büyükka Bay et al., 2010 [4] | 1 | Oligodendroglioma (low grade) |
| Ud Din et al., 2012 [31] | 4 | Oligodendroglioma (low grade) |
| Unal et al., 2014 [32] | 1 | Oligodendroglioma |
| Serrano-Arévalo et al., 2016 [25] | 1 | Oligodendroglioma (low grade) |
